# Impact of Surgical Management of Endometrioma on AMH Levels and Pregnancy Rates: A Review of Recent Literature

**DOI:** 10.3390/jcm10030414

**Published:** 2021-01-22

**Authors:** Ana Sofia Pais, Clara Flagothier, Linda Tebache, Teresa Almeida Santos, Michelle Nisolle

**Affiliations:** 1 Reproductive Medicine Unit, Centro Hospitalar e Universitário de Coimbra, 3004-561 Coimbra, Portugal; teresaalmeidasantos@chuc.min-saude.pt; 2 Obstetrics Department, Faculty of Medicine, University of Coimbra, 3000-370 Coimbra, Portugal; 3 Coimbra Institute for Clinical and Biomedical Research (iCBR) Area of Environment Genetics and Oncobiology (CIMAGO), Biophysics Institute of Faculty of Medicine, University of Coimbra, 3000-548 Coimbra, Portugal; 4 Center for Innovative Biomedicine and Biotechnology (CIBB), University of Coimbra, 3000-548 Coimbra, Portugal; 5 Clinical Academic Center of Coimbra (CACC), 3000-548 Coimbra, Portugal; 6 Department of Obstetrics and Gynaecology, Hospital CHR Liège, University of Liège, 4000 Liège, Belgium; clara.flagothier@student.uliege.be (C.F.); linteb@hotmail.com (L.T.); michelle.nisolle@chuliege.be (M.N.); 7 Faculty of Medicine, University of Coimbra, 3000-370 Coimbra, Portugal

**Keywords:** endometriosis, endometrioma surgery, ovarian reserve, anti-Müllerian hormone, spontaneous pregnancy

## Abstract

Ovarian endometrioma are found in up to 40% of women with endometriosis and 50% of infertile women. The best surgical approach for endometrioma and its impact on pregnancy rates is still controversial. Therefore, we conducted a literature review on surgical management of ovarian endometrioma and its impact on pregnancy rates and ovarian reserve, assessed by anti-Müllerian hormone (AMH) serum levels. Ovarian cystectomy is the preferred technique, as it is associated with lower recurrence and higher spontaneous pregnancy rate. However, ablative approaches and combined techniques are becoming more popular as ovarian reserve is less affected and there are slightly higher pregnancy rates. Preoperative AMH level might be useful to predict the occurrence of pregnancy. In conclusion, AMH should be included in the preoperative evaluation of reproductive aged women with endometriosis. The surgical options for ovarian endometrioma should be individualized. The endometrioma ablation procedure seems to be the most promising treatment.

## 1. Introduction

Endometriosis is an inflammatory condition characterized by the presence of endometrial-like tissue outside the uterus. It affects mostly women of reproductive age and approximately 30–50% of women with endometriosis may present infertility [1].

Between 17% and 44% of endometriosis patients have endometriotic ovarian cysts (endometrioma), which are bilateral in about 19–28% of cases [2,3]. The aetiopathogenesis of endometrioma is still uncertain and several hypotheses have emerged. Hughesdon [4] and Brosens et al. [5] demonstrated the formation of a pseudocyst by invagination of the ovarian cortex following the bleeding of a superficial endometriotic implant and the accumulation of menstrual debris. According to Nezhat et al. [6], endometrioma results from the transformation of a functional cyst. More recently, Donnez et al. [7] confirmed the involvement of metaplasia of invaginated coelomic epithelium in the origin of endometrioma.

Recommendations on the different surgical options available for ovarian endometrioma have recently been published by the working group of the European Society for Gynaecological Endoscopy (ESGE), the European Society of Human Reproduction and Embryology (ESHRE) and the World Endometriosis Society (WES) [8]. In summary, the available approaches for conservative surgical treatment of ovarian endometrioma are cystectomy, ablation or a combined technique.

Laparoscopic ovarian cystectomy is performed by the stripping technique, in which the drained endometrioma and ovarian cortex are pulled apart and haemostasis is applied on the ovarian cyst bed [9]. Traction and counter-traction must be performed using appropriate instruments with low to moderate force to avoid excessive bleeding.

In the ablative approach, endometrioma is fenestrated, drained and washed out and the cyst wall is then destroyed with an energy source, such as a CO_2_ laser, bipolar coagulation or plasma energy [9]. Care must be taken to ablate the entire surface of the cyst wall in order to reduce the risk of residual ovarian endometrioma. The entire depth of the cyst capsule must not be ablated as endometriotic tissue is present only superficially, with a mean depth of 0.6 mm [10].

In cases of large ovarian endometrioma, a three-step approach could be suggested, requiring a first laparoscopy for draining the cyst, followed by 3 months of gonadotropin-releasing hormone (GnRH) agonist therapy [7,11]. At the end of the medical treatment, a second laparoscopy is performed in order to ablate the reduced ovarian endometrioma [7,8].

In order to avoid two laparoscopic procedures, Donnez et al. [11] described a combined technique in which 80–90% of the endometrioma is excised according to the cystectomy technique, and a CO_2_ laser is then used to vaporize the remaining 10–20% of the endometrioma close to the ovarian hilus. Indeed, in this region of the ovary, dissection is usually more difficult and is associated with a higher risk of bleeding which needs coagulation close to the ovarian vessels.

Surgical treatment of endometrioma improves patients’ symptoms, such as pain, but the most appropriate approach for reproductive outcomes is still controversial, according to the Royal College of Obstetricians and Gynaecologists (RCOG) [12]. The guidelines from the ESHRE and a Cochrane review state that ovarian cystectomy is the preferred technique in terms of recurrence and spontaneous pregnancy rate after surgery [13,14]. In infertile women with stage I/II endometriosis according to the revised American Fertility Society (rAFS) classification of the American Society for Reproductive Medicine (ASRM), ESHRE recommends performing an operative laparoscopy rather than only a diagnostic laparoscopy [13]. On the other hand, ASRM proposed that in the initial stages and in women under than 35 years, expectant management or superovulation/intrauterine insemination can be considered as first-line therapy [15]. For stage III–IV disease, both societies agree with the benefit of surgical therapy [13,15]. However, the safety of this option has been questioned as it may cause ovarian damage, with a negative effect on ovarian reserve.

Ovarian reserve is defined as the functional potential of the ovary and reflects the number and quality of the follicles in the ovaries at any given time. Anti-Müllerian hormone (AMH) is a reliable marker of ovarian reserve [16]. AMH is a glycoprotein secreted by granulosa cells of primary, pre-antral and antral follicles, but it is not produced by primordial follicles. After the AMH peak at 24 years old, it gradually decreases to become undetectable at menopause [17,18].

The risk of postsurgical ovarian failure has reopened the debate between excision and ablation [19]. The deleterious effects of the presence of endometriosis in the ovarian reserve itself as well as the risk of affecting the ovarian reserve by the surgical procedures are taken into account when deciding whether or not to operate on patients who want a pregnancy [20,21,22,23]. Therefore, in many centres, patients are directly referred to in vitro fertilization (IVF) instead of offering them an appropriate surgical procedure associated with the possibility of getting pregnant spontaneously. Therefore, as endometriosis is mainly found in women of reproductive age, the impact of endometrioma and its treatment on ovarian function must be evaluated in order to maintain the best chances of pregnancy.

The aim of this review was to evaluate the effect of surgical management of endometrioma on ovarian reserve, assessed by serum AMH concentration, and on pregnancy rates, through a review of the literature.

## 2. Methods

The literature search was done using the PubMed and Cochrane search engines. The keywords used were “endometrioma”, “surgery”, “ovarian reserve”, “AMH”, “anti-Müllerian hormone” and “spontaneous pregnancy”. This research was limited to English and French language publications, focusing on the last 5 years (2015–2019). The studies were selected based on the abstract. This research was supplemented by the bibliography of experts and the references cited in the documents reviewed. Clinical cases and comments were excluded.

## 3. Results

### 3.1. AMH after Surgery for Endometrioma

#### 3.1.1. AMH and Ovarian Cystectomy

A recent systematic review and meta-analysis confirmed previous studies and systematic reviews reporting consistent evidence of a negative impact of excision of endometrioma on ovarian reserve [3,21,22]. In the late postoperative period (9 to 12 months), a 39.5% and 57% reduction in postoperative circulating AMH was observed in patients with unilateral (1.65 ng/mL, 95% confidence interval (CI) 1.15 to 2.15) and bilateral endometriomas (2.03 ng/mL, 95% CI 1.47 to 2.58), respectively [3].

Celik et al. showed that cystectomy leads to a significant and progressive decrease (61%) in serum AMH levels in a prospective study with 65 patients comparing AMH measured preoperatively (1.78 ± 1.71 ng/mL), at 6 weeks (1.32 ± 1.29 ng/mL) and 6 months after surgery (0.72 ± 0.79 ng/mL) [24]. Alborzi et al., in a prospective study with 193 patients, observed the same trend within 9 months of follow-up (baseline AMH = 3.86 ± 3.58 ng/mL; 9 months after surgery = 1.77 ± 1.76 ng/mL) [25].

Subsequent studies assessing AMH levels up to 1 year after surgery revealed that this decrease would be only temporary and could recover [26,27,28,29,30]. Vignali et al., in a prospective study with 22 patients undergoing laparoscopic cystectomy for endometrioma, verified that the mean 1-year postoperative AMH levels were not statistically different from the mean values prior to surgery [26]. Sugita et al. also performed a prospective study including 39 patients and observed that 50% had higher AMH levels 1 year after surgery than 1 month after surgery (20 vs. 19 patients). The comparison of these two groups (increase vs. decrease in AMH levels at 1-year follow-up), showed a significant difference in the number of follicles in specimens due to removal of ovarian cortex during surgery [27]. A larger prospective study with 171 patients performed by Wang et al. showed that, 12 months after surgery, AMH levels were no different from the preoperative assessment in small cysts (≤7 cm), unilateral cysts and stage III endometriosis [28]. Kostrzewa et al. also performed a 1-year follow-up and observed a significant decrease in AMH levels 3 months after cystectomy (4.89 ± 3.66 vs. 3.45 ± 3.37 ng/mL, *p* < 0.001), but no further fall in the 1-year assessment (3-months = 3.45 ± 3.37 vs. 1-year = 3.43 ± 3.62 ng/mL, *p* > 0.05) [29]. The same result was achieved by Kovačević et al. in a prospective study that enrolled 54 patients (37 with unilateral endometrioma and 17 with bilateral) [30].

In a prospective cohort study with 59 patients with endometrioma and 16 with other benign cysts, the comparison of the postoperative decline in serum AMH revealed a higher and significant decrease in the group with endometrioma (baseline = 4.3 ± 0.4 vs. 3 months after surgery = 2.8 ± 0.2 ng/mL, *p* < 0.001) [31]. The same result was achieved by Taniguchi et al. in a study that enrolled 40 women with endometrioma and 16 with benign ovarian tumours. The postoperative decline rate of AMH levels had statistically significant differences at 6 months after surgery when patients with endometrioma were compared with those with other ovarian cysts (0.63 (0.26–0.69) vs. 0.24 (–0.86–0.32), *p* < 0.05). However, in the evaluation performed 1 year after surgery, that reduction did not remain significant (0.46 (0.14–0.73) vs. 0.21 (–0.52–0.78), *p* = 0.34) [32]. Kostrzewa et al. also compared a group of patients with endometrioma (*n* = 35) with a group with other benign ovarian tumours (*n* = 35). The decline in serum AMH levels in the first 3 months following surgery was 3 times higher following laparoscopic cystectomy of endometrioma (45.39% vs. 14.87%; *p* = 0.021) [29]. The same result was observed when comparing laparoscopic cystectomy in patients with endometrioma and dermoid cysts [33].

The reduction in AMH level after surgery is higher in bilateral endometrioma [3,24,26,27,28,34]. Additionally, it is inversely correlated with the diameter of the cyst, with a clear decrease when the cyst is greater than 5 cm. Nevertheless, this decline is not associated with the follicular loss evaluated by histology [24,28,35].

Kim et al. reported that the decrease in AMH levels was also dependent on the stages of endometriosis, with stages III and IV having a significantly greater decrease in AMH from the pre- to postoperative period in comparison with lower stages [28,31]. However, they showed that the decline was independent of multiplicity, bilaterality and GnRH agonist use [31]. In addition, the postsurgical reduction in patients over 35 years old was greater, highlighting the negative effect of age on ovarian function [32].

In a prospective controlled study, Muzii et al. observed that surgery for recurrent endometriomas is more harmful to ovarian reserve, even though they only used antral follicle count (AFC) and ovarian volume [36].

Recently, in a prospective study with 124 patients, Zhou et al. verified that a decrease in AMH levels after surgery happened in both patients with high (>2 ng/mL) and low (≤2 ng/mL) preoperative AMH levels (4.51 ± 1.20 vs. 3.04 ± 0.90 ng/mL, *p* < 0.001; 0.89 ± 0.36 vs. 0.51 ± 0.27 ng/mL, *p* < 0.001, respectively) [37].

The presurgical identification of patients with decreased ovarian reserve and the risk of poor postoperative ovarian response can be predicted using preoperative measurements of serum AMH. Ozaki et al. proposed that 2.1 ng/mL was the best cut-off value of preoperative AMH for predicting diminished ovarian reserve (DOR) at 3 and 6 months in patients undergoing unilateral cystectomy. In cases of bilateral ovarian surgery, the optimal cut-off points were 3.0 ng/mL to predict DOR 3 months after surgery and 3.5 ng/mL to predict DOR 6 months after surgery [34].

After complete excision of the cyst capsule, final hemostasis must be guaranteed [8]. The traditional hemostatic technique is bipolar coagulation, but it might be used with caution to avoid excessive compromise of ovarian reserve [8,38]. A recent systematic review and meta-analysis comparing this approach with suture, ultrasonic energy and intra-ovarian hemostatic sealants showed a lower impact in postoperative AMH levels with the use of suturing [39]. The same result was achieved in a previous meta-analysis comparing just bipolar coagulation vs. suture [40]. In this field, there are two ongoing randomized clinical trials comparing ovarian function after laparoscopic cystectomy for endometrioma complemented with hemostatic approaches, the results of which are highly anticipated [41,42]. Dual-wavelength laser systems (DWLS) are a new instrument that have been described for hemostasis and that seem not to determine a significant reduction of ovarian reserve [43].

#### 3.1.2. AMH and Endometrioma Ablation

Roman et al. conducted a prospective study [44] analysing serum AMH levels at 3 time points (before surgery, 3 months after surgery and >6 months after surgery) in 22 patients with unilateral endometrioma ≥30 mm without any history of previous surgery who underwent ablation with vaporization using plasma energy. This resulted in a postoperative drop in the AMH level, followed by a gradual re-increase. There was usually no return to preoperative AMH values, but the difference no longer reached statistical significance >6 months after surgery.

A more recent study by Stochino-Loi et al. [45] gathering 180 patients with stage III and IV endometriosis and intention of pregnancy compared AMH evolution after plasma energy vaporization according to preoperative AMH levels ≥ or <2 ng/mL. Plasma energy ablation caused a temporary decrease in AMH level in both groups.

#### 3.1.3. Comparison of AMH after Ovarian Cystectomy and Endometrioma Vaporization

Saito et al. [46] performed a prospective study comparing AMH levels after cystectomy or endometrioma vaporization with bipolar current forceps in unilateral and bilateral endometrioma. They demonstrated that both methods decrease the ovarian reserve, especially in cases of severe endometriosis or over the age of 38. However, the postoperative decline in AMH was higher after cystectomy than vaporization and was statistically significant for bilateral endometrioma. In patients submitted to bilateral cystectomy, AMH levels declined from 3.1 ± 1.7 ng/mL at preoperative staging to 0.5 ± 0.5 ng/mL at 1 month after surgery, 0.8 ± 0.7 ng/mL at 6 months and 0.8 ± 0.7 ng/mL at 1 year. For bilateral vaporization, preoperative AMH levels were 2.7 ± 1.8 ng/mL and decreased to 0.8 ± 0.6 ng/mL at 1 month after surgery, 1.2 ± 1.3 ng/mL at 6 months and 1.3 ± 1.5 ng/mL at 1 year [46].

The multicentre randomized clinical trial of Candiani et al. [47] compared changes in AMH and antral follicle count (AFC) after cystectomy or CO_2_ laser vaporization in 60 patients with endometrioma larger than 3 cm. Three months after surgery, they observed a significant decrease in serum AMH in the subjects treated with cystectomy (from 2.6 ± 1.4 to 1.8 ± 0.8 ng/mL; 95% CI: −1.3 to −0.2; *p* = 0.012), while no significant reduction was evident in the group treated with CO_2_ laser vaporization (from 2.3 ± 1.1 to 1.9 ± 0.9 ng/mL; 95% CI: −1 to −0.2; *p* = 0.09).

A retrospective study with prospective recording of data performed by the same group showed that postoperative recurrence rates were comparable between patients that underwent CO_2_ fiber laser vaporization or cystectomy [48]. During a 3-year follow-up, no difference was observed in recurrence of ovarian endometriosis (cystectomy group = 6.3% vs. vaporization group = 4.9%, *p* = 0.74) and of endometriosis-related pain (cystectomy group = 7.8% vs. vaporization group = 9.8%, *p* = 0.67).

For large endometriomas (>5 cm), a prospective randomized study performed by Giampaolino et al. revealed that the decrease of AMH levels assessed 3 months after surgery was greater following excisional surgery than ablative treatment (−24.1 ± 9.3% vs −14.8 ± 6.7%, *p* = 0.011) [49].

#### 3.1.4. AMH and the Combined Technique

The combined approach, using excision of 80–90% of the cyst and ablation of the rest, has been proven not to be deleterious to the ovary through comparison of the ovarian volume and AFC. AMH serum levels were not analysed in this study published by Donnez et al. [11].

Tsolakidis et al. [50] performed a prospective randomized clinical trial comparing AMH levels before and 6 months after laparoscopic cystectomy for endometrioma or the three-step procedure. They found that AMH is less diminished after the three-step procedure (from 4.5 ± 0.4 to 3.99 ± 0.6 ng/mL, *p* > 0.05) compared with cystectomy of endometrioma (from 3.9 ± 0.4 to 2.9 ± 0.2 ng/mL; *p* = 0.026). This is explained by the fact that vaporization avoids ovarian tissue ablation and excessive thermal damage.

### 3.2. Pregnancy Rate after Surgery for Endometrioma

The role of surgery to improve the pregnancy rate in infertile women with endometriosis is controversial.

In a retrospective study with 43 infertile women with surgically proven endometriosis and no other factors, Lee et al. reported that the spontaneous conception rate was 41.9% during the first year after laparoscopic surgery, which involved the destruction or removal of all visible endometriotic implants and the lysis of adhesions [51].

For endometrioma, surgery seems to improve the success rates of fertility treatment by between 20% and 60% [9,52,53,54]. A recent meta-analysis compared pregnancy rates based on four different treatments for endometrioma in infertile women: surgery (excisional and/or ablative) + assisted reproductive technology (ART), surgery + spontaneous pregnancy, aspiration ± sclerotherapy + ART and only ART. There was no difference among groups. However, the success rate of surgery was higher (43.8%, confidence interval (CI): 22.5–66.4), while the success rate of only ART was the lowest (32%, CI: 15.0–52.0) [55].

#### 3.2.1. Pregnancy Rate and Ovarian Cystectomy

In a meta-analysis by Vercellini et al., the chance of pregnancy after laparoscopic excision of endometriomas ranged from 30% to 67%, with an overall weighted mean of about 50% [56]. Zhou et al. recently conducted a prospective study with 124 patients that was consistent with these results, with a total spontaneous pregnancy rate of 50.49% within 24 months after excisional surgery [37]. Taniguchi et al. also reported a cumulative pregnancy rate of 50% after cystectomy for ovarian endometrioma [32].

Women with higher AMH levels had a significantly higher cumulative pregnancy rate after surgery for endometrioma [37,54,57]. Ozaki et al. compared patients according to preoperative ovarian reserve and observed that the rate of spontaneous pregnancy was greater in patients with AMH ≥ 1.1 ng/mL (59.2% vs. 14.3%, *p* = 0.04) [34]. According to Dong et al., the best cut-off point of the preoperative AMH for postoperative spontaneous pregnancy is > 3.68 ng/mL (Hazard ratio (HR): 2.383; 95% CI, 1.093–5.197) [57]. A very similar value was proposed by Zhou et al. (3.545 ng/mL; sensitivity 80.39%; specificity 69.23%) [37]. Thus, preoperative AMH level might be a useful marker to predict the occurrence of natural pregnancy and could be offered as part of patient assessment [37].

Studies comparing AMH level after cystectomy between patients who became pregnant and those who did not showed a higher AMH level 1 year after surgery in the group of pregnant women [32,58].

When the likelihood of spontaneous pregnancy after laparoscopic cystectomy of endometriomas was compared with other benign ovarian cysts, it was observed that it is more than 3 times higher in the group of patients with other benign tumours (HR 3.57; *p* = 0.03) [29].

#### 3.2.2. Pregnancy Rate and Endometrioma Ablation

A retrospective pilot study by Roman et al. [59] evaluated recurrence and pregnancy rates in 55 patients with endometrioma treated by ablation using plasma energy. Recurrence (10.9%) and pregnancy rates (67%, spontaneously in 59%) are encouraging and are comparable to the reported results after endometrioma cystectomy. A more recent study by the same group enrolled 22 patients with unilateral endometrioma ≥30 mm and no history of previous surgery who underwent ablation with vaporization using plasma energy. The overall pregnancy rate during postoperative follow-up reached 73% [44].

Stochino-Loi et al. [45] performed a retrospective comparative study with 180 patients with stage III and IV endometriosis and pregnancy intention. They observed that the probability of postoperative pregnancy was comparable between women with low and normal AMH levels (AMH levels < 2 ng/mL = 73.9% and AMH levels ≥ 2 ng/mL = 74.6%); most of them got pregnant spontaneously (58.8% and 54%, respectively).

Motte et al. [60] conducted a retrospective case control study in which plasma energy ablative therapy demonstrated a higher implantation, pregnancy and delivery rates per IVF cycle, albeit with a lower number of oocytes retrieved. Thus, plasma energy has been suggested as a more favourable ablative technique for endometrioma management.

#### 3.2.3. Comparison of Pregnancy Rate after Ovarian Cystectomy and Endometrioma Vaporization

A Cochrane review by Hart et al. published in 2008 showed a beneficial effect of excisional surgery over drainage or ablation of endometrioma when considering achievement of spontaneous pregnancy in subfertile women (odds ratio (OR) 5.21, CI 2.04–13.29) [14]. However, there were only two studies that addressed this issue, so publication bias cannot be excluded.

#### 3.2.4. Pregnancy Rate and the Combined Technique

In a descriptive and prospective study, Donnez et al. reported a pregnancy rate of 41% at a mean follow-up of 8.3 months after the combined approach for endometrioma [11].

## 4. Discussion

The benefit of endometrioma excision for pain management is consensual, but surgical excision for the sole purpose of improving reproductive outcomes is controversial [9]. Ovarian involvement with endometriosis might have a negative impact on ovarian reserve [23,26,32]. That fact, alongside the risk of postsurgical ovarian failure, has reopened the debate between excision and ablation [19].

The reduction of ovarian reserve after surgery for endometrioma is inevitable, regardless of the technique. Both excisional and ablative approaches lead to a postsurgical decrease of up to 60% in AMH levels. However, studies comparing the two techniques show a higher and significant decrease after cystectomy [46,47].

The decline in ovarian reserve after ovarian surgery is multifactorial. Healthy ovarian tissue may be unintentionally removed during ovarian cystectomy due to the absence of a clear histologic cleavage plane, which can result in loss of follicles. This justifies the theory that ovarian reserve is better preserved by ablation than by cystectomy. However, other proposed mechanisms for the ovarian reserve decline include thermal damage caused by bipolar coagulation, ovarian vascular injury and postoperative inflammatory response [19,24,28,61]. Therefore, bipolar electrocoagulation should be kept to a minimum, especially for patients with reproductive goals [61]. With the use of a CO_2_ laser, the glandular epithelium and the underlying stroma [11,47] are destroyed without reaching the fibrous capsule surrounding the endometrioma or healthy neighbouring ovarian cortex. The CO_2_ laser would provide better control of the depth of vaporization, remaining superficial compared to bipolar electrocoagulation [47,50]. This is an advantage as it would not be necessary to destroy the entire fibrous capsule by vaporization, as only 1.0–1.5 mm of the inner lining would be sufficient [62]. The CO_2_ laser as well as plasma energy are techniques for sparing ovarian tissue with a shallower thermal diffusion [50,59]. Their low thermal energy avoids excessive ischaemic damage while providing high precision and optimal coagulation, reducing the need for electrocoagulation or suturing [19,50]. Thus, CO_2_ technology may be used to treat endometrioma with minimal damage to the adjacent healthy ovarian tissue and it might be an alternative treatment in women with a desire for pregnancy.

Excision of the ovarian cortex could be involved in the reduction of ovarian reserve just after surgery, but a continuous decrease could be attributed to other factors, such as vascular compromise by excessive coagulation or adhesiolysis as well as postsurgical inflammation [24,26,27,44].

The number of studies that have evaluated changes in ovarian reserve after cystectomy over a period longer than 6 months is limited, but it seems that the decrease in AMH following surgery for endometrioma is temporary and can be recovered. This can be explained by surgery-related reversible mechanisms related to ovarian vasculature and inflammation-mediated injuries. After ovarian injury, compensatory mechanisms may include the recruitment and growth of primordial follicles and the excessive activation of granulosa cells [28]. This leads to rearrangements of the cohort of follicles, including follicles producing AMH, which can explain the “recovery” in the ovarian reserve. The delay in this recovery is explained by the approximate 180-day duration of folliculogenesis from the primordial follicles to the pre-ovulatory follicles [27]. A similar pattern of AMH recovery has been reported in young women after chemotherapy, in which a complete restoration of AMH levels was observed [26,63]. However, some studies showed that ovarian reserve cannot be fully restored in all patients after surgery for endometrioma, indicating some elements of permanent damage. Since the literature on the late postoperative period is scarce, recovery of the ovarian reserve should be interpreted with caution [3]. Furthermore, factors like AMH decline with age and endometriosis must be considered [23,64].

Bilaterality, size of endometrioma, stage of endometriosis and patient’s age are independent factors that should be also considered when planning a surgery in patients who are interested in preserving their fertility [3,30]. Bilateral endometriomas, stage III/IV endometriosis and patients over the age of 35 have a higher impact on postsurgical AMH levels. For large cysts, a proportional loss of healthy ovarian tissue with the diameter of the cyst can explain the higher decrease of AMH levels [35,65]. Additionally, for endometriomas with more than 5 cm, ablative treatment seems to have low impact in postoperative AMH levels than excisional surgery [49]. For recurrent endometrioma, a second surgery is associated with higher loss of ovarian tissue and is more harmful to the ovarian reserve [36]. Indications for surgery for recurrent endometrioma should thus be considered with caution and excisional surgery must be avoided [36]. Medical treatment may be the first option, but when surgery may still be indicated, an ablative approach can be considered, as recurrence rates are similar [48].

All of these factors will allow clinicians to select therapies to prevent further decline of ovarian reserve, especially for infertile patients with ovarian endometrioma [34]. Therefore, surgery should be performed when mandatory, such as pain refractory to medical therapy, pain associated with otherwise unexplained infertility and in the case of non-reassuring features of the cyst on preoperative ultrasound [66]. Individual reproductive plans and oocyte or ovarian tissue cryopreservation should be discussed with patients before surgery. Ideally, surgery can be postponed until the reproductive project is complete [3].

The decline of AMH levels after surgery is higher in patients with ovarian endometrioma than in those with other benign tumours [29,31,32,33]. This is in line with the impact per se of endometriosis in ovarian reserve and the fact that it is also present when surgery is performed by a specialised surgeon [26,32,66]. The likelihood of spontaneous pregnancy after surgery is also lower in patients with endometrioma [29].

According to the ESHRE guideline, there is evidence to suggest that ovarian cystectomy via stripping is the preferable surgical technique for management of endometrioma, compared with other excisional/ablative techniques in terms of the pregnancy rate [13,14]. However, studies carried out later show a higher overall pregnancy rate after the ablative approach than the excisional (67–73% vs. 30–67%) [44,59]. This fact is in line with the lowest impact on AMH levels.

Favourable preoperative ovarian reserve and its postoperative maintenance together may be implicated in postsurgical pregnancy after surgery for endometrioma [34,37]. The potential risk of postsurgical poor ovarian response could be predicted by using optimal cut-off points of presurgical AMH levels (2.1 ng/mL of unilateral endometrioma; 3 and 3.5 ng/mL for bilateral endometrioma at 3 and 6 months after surgery, respectively) [34]. The cut-off value to predict spontaneous pregnancy rates after endometrioma cystectomy is approximately 3.5 ng/mL, with higher AMH levels associated with a higher pregnancy rate [37,54,57]. Thus, after cystectomy, better ovarian reserve with optimal rearrangement of the follicle cohort may be related to subsequent pregnancy [58]. In patients at risk, alternative management of cystectomy should be foreseen. However, AMH is a quantitative but not qualitative surrogate for oocytes [34].

In patients with stage III and IV endometriosis submitted to ablative surgery, the probability of pregnancy and the risk of decreasing ovarian reserve is similar in patients with high and low preoperative AMH levels [45]. Therefore, a young patient suffering from severe endometriosis with a decreased ovarian reserve and a preoperative AMH level below normal could benefit from surgical management. This surgery could restore the capacity of spontaneous pregnancy in this population and may be an alternative to ART [45].

This review highlights the importance of preoperative evaluation of AMH in the therapeutic planning of patients with endometrioma and in the selection of the surgical technique. Based on this value, it is possible to offer more detailed preoperative counselling regarding the pregnancy rate after surgery and the risk of decreased ovarian reserve, assessed through AMH values. Recent studies suggest that the ablative approach, namely, with the use of a CO_2_ laser, seems to be the most interesting surgical technique, with the least impact on postoperative AMH levels and better pregnancy rates. However, this review has some limitations as more studies, namely, randomized clinical trials, are needed to draw definitive conclusions. Additionally, more studies assessing live birth rate rather than pregnancy rate are needed, as live birth rate was recently defined as a core outcome set for endometriosis [67].

## 5. Conclusions

In conclusion, measurement of AMH should be included in the evaluation of reproductive-age women with endometriosis. The indication of surgery for an ovarian endometrioma should be thoroughly discussed with the patient, with particular emphasis on the issue of possible damage to the ovarian reserve. The review of the literature demonstrates that the endometrioma ablation procedure, even if performed in patients with a decreased ovarian reserve, is beneficial in terms of pregnancy.
